# Cell surface profiling of cultured cells by direct hydrazide capture of oxidized glycoproteins

**DOI:** 10.1016/j.mex.2023.102349

**Published:** 2023-08-26

**Authors:** Tammy-Lynn Tremblay, François Fauteux, Deborah Callaghan, Jennifer J. Hill

**Affiliations:** Human Health Therapeutics, National Research Council of Canada, 100 Sussex Dr., Ottawa, Ontario K1A0R6, Canada

**Keywords:** Glycoproteomics, Cell membrane proteins, Mass spectrometry, Surfaceome, Direct Cell Surface Capture (D-CSC)

## Abstract

Glycoproteins are a particularly interesting subset of the cellular proteome as a high proportion of proteins present on the extracellular cell surface are glycosylated. These cell surface proteins are ideal targets for biologic drug therapies or for diagnostics tests. Here, we describe a modification of the well-described Cell Surface Capture (CSC) method for the selective isolation and identification of cell surface glycoproteins that contain N-linked carbohydrates. This modification, which we refer to as Direct Cell Surface Capture (D-CSC), is based on oxidation of cell surface glycans on intact cells, followed by direct conjugation of the oxidized oligosaccharides to a solid support using hydrazide chemistry, with no biotinylation step. As a proof-of-principle, we applied D-CSC to the analysis of cell surface membrane proteins of three adherent cancer cell lines (A549, OVCAR3, and U87MG) and compared our results to those published using the well-established Cell Surface Capture (CSC) method, demonstrating comparable selectivity for cell surface proteins.

• A method enabling the identification of cell surface proteins from cells in culture is described.
• Application of this method to profile the cell surface on three different cancer cell lines is included.

## Graphical abstract

**Figure:**
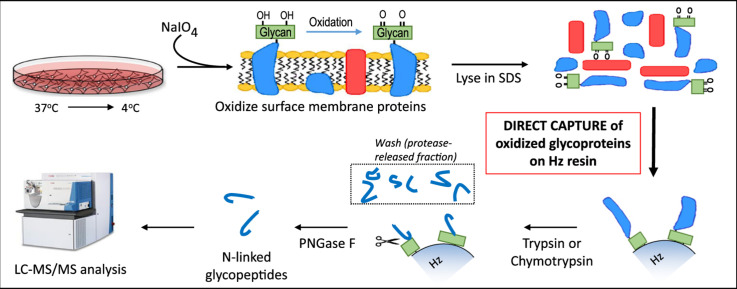

Specifications table

**Table:** 

Subject area:	Biochemistry, Genetics and Molecular Biology
More specific subject area:	Glycoproteomics of cell surface proteins
Name of your method:	Direct Cell Surface Capture (D-CSC)
Name and reference of original method:	Cell Surface Capture (CSC) Wollscheid, B.; Bausch-Fluck, D.; Henderson, C.; O'Brien, R.; Bibel, M.; Schiess, R.; Aebersold, R.; Watts, J.D. Mass-spectrometric identification and relative quantification of N-linked cell surface glycoproteins. *Nat. Biotechnol.***2009**, *27*, 378–386, doi:10.1038/nbt.1532
Resource availability:	Fragpipe - https://fragpipe.nesvilab.org/

## Method details

The method described here, which we refer to as Direct Cell Surface Capture (D-CSC), is a modified version of the previously published and highly utilized Cell Surface Capture (CSC) method [2,8] that incorporates aspects of hydrazide-bead based glycocapture workflows [3,^6^,9]. In the D-CSC protocol, glycans on cell surface proteins are modified by oxidation with sodium meta-periodate and the resulting aldehyde groups are used to immobilize these modified glycoproteins by the formation of a covalent bond between the aldehyde and a hydrazide group on a gel-based bead. Glycoproteins immobilized on the bead are subsequently digested with trypsin or chymotrypsin and then peptides containing an N-linked glycosylation site are released using PNGaseF. These N-glycopeptides are then analyzed by mass spectrometry to identify cell surface proteins. The original published CSC method [8] also oxidizes glycans on the cell surface, but then reacts the resulting aldehydes with a modified biotin (biocytin hydrazide) molecule. Proteins are then digested with trypsin and biotinylated peptides are immobilized on streptavidin resin prior to release with PNGaseF. While the biotinylation step appears to be added to improve the cell surface selectivity of modification, our results suggest that direct capture of oxidized glycoproteins is equally selective for cell surface proteins.

**Glycan oxidation of cell surface proteins**

Material and equipment required for 2 × 150 mm plates of adherent cells:

- Phosphate-buffer saline (PBS), pH 6.5 (adjusted if needed) and chilled to 4 °C
- Oxidation solution: 10 mM Sodium meta periodate (NaIO_4_)
  ○ Place 0.086 g of NaIO_4_ in a 50 mL conical tube covered with aluminum foil.
  ○ Immediately prior to use, add 40 mL of cold PBS pH 6.5.
- 10X Quenching solution: 200 mM Sodium Sulfite (Na_2_SO_3_)
  ○ Place 0.252 g of Na_2_SO_3_ in a 15 mL conical tube.
  ○ Immediately prior to use, add 10 mL of cold PBS pH 6.5.
- Large container filled with crushed ice in which 2 plates can fit side by side
- 15 and 50 mL plastic conical tubes
- Aluminum foil
- Timer
- 1 mL pipette and tips
- Cell scraper
- Refrigerated swinging-bucket centrifuge capable of centrifuging 15 and 50 mL conical tubes.

Procedure:

1. Grow the desired cells to approximately 80% confluency.
  *Note: Typically, 20–30 million cells are adequate for good results. We often use 2 large (150* *mm) plates to obtain > 1* *mg of total protein for adherent cell lines. However, the number of plates might need to be increased depending on cell type.*
2. For adherent cell lines, place the cell culture plates on a flat bed of crushed ice to inhibit cellular trafficking of cell membrane proteins. For suspension cell lines, gently pellet the cells in a 50 mL conical tube by centrifugation at 200 g for 5 min at 4 °C and place the tube on ice.
  *Note: This protocol describes the cell surface labeling of adherent cell lines while they are still attached to the plate. This method has the advantage of maintaining high cell viability and integrity, but may bias surface profiling results to the exposed apical surface. If desired, a soft detachment method suitable to the adherent cell line can be used at this stage to put adherent cells into suspension while maintaining cell viability. Detached adherent cells can be treated as a suspension cell line for the remainder of this protocol. Detachment may result in increased labeling of the basal cell surface and increased identification of basal surface proteins. Detachment may lead to loss of some surface receptors, especially if an enzymatic detachment method is used, and some contamination with intracellular ER or Golgi-resident glycoproteins may occur if membrane integrity is disrupted during detachment.*
3. To expose the cells to 10 mM NaIO_4_, carefully remove the cell media and slowly add 18 mL of cold Oxidation solution to each plate/tube.
4. Protect from light by covering with foil and incubate for 10 min on ice rocking plates every 2 min to ensure the cells do not dry out. It is generally not necessary to rock tubes.
5. Stop the oxidation reaction by adding 1900 μL of cold 10X Quenching solution to each plate/tube for a final concentration of ∼20 mM Na_2_SO_3_.
6. Mix gently by rocking each plate/tube a few times and then incubate for an additional 6 min on ice. Rock plates every 2 min to ensure the cells do not dry out.
7. For suspension cells, skip to Step 8. For adherent cells on plates:
  a. Transfer 8 mL of the liquid from each plate into a single 50 mL plastic tube placed on ice. This solution may contain some detached cells.
  b. Using a cell scraper, scrape the cells into the remaining buffer and transfer those cells into the same 50 mL tube.
  c. Add an additional 5 mL of cold PBS pH 6.5 to each plate. Swirl to re-suspend any remaining cells then combine the cell suspension into the 50 mL conical tube as well.
8. Pellet the cells by centrifugation at 400 g for 5 min at 4 °C, then carefully remove the supernatant.
  *Note: Centrifugation speed and time should be adjusted as needed for each cell line.*
9. *Re*-suspend the cells in 10 mL cold PBS, pH 6.5 and transfer into a 15 mL tube.
10. Pellet the cells by centrifugation at 400 g for 5 min at 4 °C.
11. Remove PBS and proceed directly with cell lysis or freeze cell pellet at −80 °C.

**Cell Lysis**

Material and equipment:

- Hydrazide (Hz) Coupling buffer: 100 mM NaOAc, 150 mM NaCl, pH 5.5)
  ○ Add 6.8 g of sodium acetate trihydrate (C_2_H_9_NaO_5_) and 4.4 g of NaCl into 450 mL of H_2_O.
  ○ Adjust pH to 5.5 with acetic acid
  ○ Adjust volume to 500 mL with H2O
  ○ Sterilize with 0.22 filter unit.
  ○ Store at room temperature (RT) for up to 1 year.
- Lysis Buffer: 0.5% SDS in Hz coupling buffer with 1/200 of protease inhibitor cocktail (Sigma, cat# P8340) and 1/1000 benzonase (Sigma, Cat# 71,205–3)
  ○ Add 0.025 g of SDS in 5 mL of Hz Coupling buffer in a 15 mL conical tube.
  ○ Immediately prior to use, add 1/200 of protease inhibitor cocktail and 1/1000 benzonase into the required volume of Lysis Buffer (see below).
- 15 mL plastic conical tubes
- 1 mL pipette and tips
- 3 mL syringe with a 33 gage needle
- MACSMix head-over-head shaker (Miltenyi Biotec) or a similar tube rotator
- Swinging-bucket centrifuge capable of centrifuging 15 mL and 50 mL conical tubes.
- Detergent Compatible (DC) protein assay kit (Bio-Rad)

Procedure:

1. Add 1 to 5 mL of Lysis Buffer to the cell pellet to lyse the cells.
  *Note: The volume of Lysis Buffer is adjusted according to the pellet size. Use a volume approximately equivalent to 10X of the pellet size (i.e. for a cell pellet with a volume around 200*μ*L, use 2* *mL of Lysis Buffer.*
2. Homogenize the cells by passing the sample through a 3 mL syringe 5 times. Place sample tube the rotator at RT for 30 min to 2 h until all the DNA has been degraded by the benzonase (until the sample is no longer viscous).
  *Note: Use a syringe with a 33 gage needle to completely aspirate and eject the cell lysate 5X. It is also possible to sonicate at this step to break up the DNA, possibly eliminating the need for benzonase treatment. However, we have found it difficult to control foaming of the SDS-based buffer during sonication.*
3. Centrifuge for 10 min at RT at 4500 g to pellet any cell debris.
4. Transfer the cleared cell lysate into a new 15 mL conical tube.
5. Quantify protein content of the cleared cell lysate using the Detergent Compatible (DC) protein assay (Bio-Rad).
6. Adjust cell lysate to a protein concentration between 1 and 1.3 mg/mL by diluting sample with additional Lysis Buffer.
7. Proceed directly with the hydrazide capture of oxidized glycoproteins, or aliquot and freeze the cell lysate at −80 °C until ready to proceed.

**Hydrazide (Hz) capture of oxidized glycoproteins**

Material and equipment:

- Affi-Gel^Ⓡ^ Hz Hydrazide Gel (Biorad, Cat# 153–6047)
- Hydrazide (Hz) Coupling buffer: 100 mM NaOAc, 150 mM NaCl, pH 5.5)
- Urea Buffer: 8 M Urea, 0.4 M ammonium bicarbonate
  ○ Place 19.2 g of Urea and 0.6 g of ammonium bicarbonate in 50 mL conical tube and add H_2_O until volume reaches the 40 mL mark.
- 50 mM AMBIC: 50 mM Ammonium Bicarbonate
  ○ Dissolve 0.16 g of ammonium bicarbonate in 40 mL of dH_2_O.
- 10 mM Dithiothreitol (DTT)
  ○ Add 0.015 g of DTT in a 15 mL conical tube and add 10 mL of 50 mM AMBIC
- 25 mM Iodoacetamide (IAA)
  ○ Add 0.046 g of IAA in a 15 mL conical tube and add 10 mL of 50 mM AMBIC
- Trypsin Digestion Solution: 50 mM AMBIC - 15% Acetonitrile (ACN)
  ○ Pipette 6 mL of Acetonitrile into a 50 mL plastic tube.
  ○ Bring volume up to the 40 mL mark with dH_2_O.
  ○ Add 0.16 g of Ammonium Bicarbonate.
- Acetonitrile (ACN)
- 1.5 M NaCl
  ○ Dissolve 43.83 g of NaCl into 500 mL of H_2_O.
  ○ Keep at RT for up to 1 year.
- Organic Wash Solution: 60% Acetonitrile (ACN)- 0.1% Trifluoroacetic acid (TFA)
  ○ Mix 8 mL of ACN with 12 mL of H_2_O.
  ○ Add 20 μL of TFA.
- 100% Methanol (MeOH)
- Sequencing grade Trypsin (Promega, Cat# V5113) and/or Chymotrypsin (Sigma, Cat# C3142)
- PNGase F (Sigma, Cat# F8435)
- 1.5 and 2 mL tubes
- Pierce centrifuge columns 0.8 mL (Thermo Fisher Scientific, Cat# 89,868), caps and end caps (Bio-Rad, #731–1660)
- Pipettes and tips
- Gel loading tip (200 μL)
- Parafilm
- MACSMix head-over-head shaker (Miltenyi Biotec) or a similar tube rotator
- Benchtop centrifuge
- Heat bloc and/or incubator
- Speed Vac concentrator
- Vacuum manifold (optional)

Procedure:

Preparation of Affi-Gel ^Ⓡ^ Hz Hydrazide Gel (Hz beads):

1. With a clean razor blade or scissors, cut off the end of a 1000 μL pipette tip to slightly widen the opening.
*Note: Hz beads are very fragile; a widened tip helps to avoid damaging the beads when pipetting.*
2. Accounting for all samples, transfer well re-suspended Hz bead solution into a 1.5 mL tube so that there are 50 μL worth of settled Hz beads for each mg of protein that will be processed, plus a little extra.
3. Centrifuge the Hz beads at 100 g for 1 min, then carefully pipet out the supernatant avoiding the pelleted Hz beads.
4. Add 1 mL of Hz coupling buffer and re-suspend the Hz beads by gently flicking the tube and by inverting it a few times.
*Note: Hz beads should never be vortexed.*
5. Repeat step 3 and 4 three more times to thoroughly wash the Hz beads.
6. Aliquot enough washed Hz beads in new 1.5 mL tubes for each sample to be processed.
  *Note: Typically, Hz capture of 1* *mg of oxidized cell lysate works well, requiring 50* μ*L of settled Hz beads per sample. However, this amount can be adjusted depending on the amount of sample to be processed.*
7. Add 1 mL of Hz coupling buffer to each tube for a final wash and let the Hz beads settle by gravity.

Glycoprotein capture:

8. With a 200 μL gel loading tip, remove the solution from each tube of Hz beads.
  *Note: The opening of gel loading tips are usually too small for the Hz beads to enter which allows the removal of most of the wash solution.*
9. Add the oxidized cell lysate prepared in the “Cell lysis” section above to each tube of semi-dry Hz beads.
  *Note: Typically, Hz capture is done with 1* *mg of oxidized cell lysate. However, good results have been obtained with lower amounts (i.e.: 500* μ*g) of cell lysate. Additionally, higher amounts (i.e. 2* *mg) of cell lysate can be used as long as the amount of washed Hz beads is adjusted accordingly. Wash times and volumes may need to be increased when using larger volumes of Hz beads to ensure thorough removal of SDS.*
10. *Re*-suspend the Hz beads by gentle flicking and/or inversion.
11. Incubate at RT on a rotator O/N (12 to 18 h).

Removal of the unbound proteins and washes:

12. For each sample, label and place a mini-spin column in a 2 mL tube that has also been labeled.
13. Centrifuge samples at 100 g for 1 min to pellet the Hz beads.
14. Carefully remove the supernatant containing the unbound proteins then add 500 μL of Urea Buffer to the Hz beads.
15. Gently re-suspend the Hz beads in Urea Buffer by pipette-mixing using a widened tip, then transfer the Hz beads into the empty mini spin column.
16. Using a vacuum manifold, wash the Hz beads 8 times with 500 μL of Urea Buffer and then 4 times with 500 μL of 50 mM AMBIC.
  *Note: If a vacuum manifold isn't available, perform the washes of the Hz beads using a centrifuge instead. Remove the washing solutions by centrifuging the mini-spin column placed in a 2* *mL tube at 100* *g for 15 s. Discard the eluted wash solution from the 2* *mL tube between washes.*

Reduction/Alkylation:

17. After the final wash, plug the bottom of the spin column so it won't leak and add 500 μL of 10 mM DTT to the Hz beads.
18. Cap the top of the column and invert it a few times to re-suspend the Hz beads in the DTT solution.
19. Incubate the column placed in 2 mL tubes in a heat block (preferred) or an incubator set at 56 °C for 1 hour.
20. Remove plug and cap (keep aside) and remove DTT by centrifugation at 100 g for 15 s.
21. Plug the bottom of the column, add 500 μL of 25 mM IAA and cap the spin column again.
22. Invert the column a few times to mix and incubate at RT for 1 hour in the dark.
23. Remove the bottom plug and cap and spin out IAA by centrifugation at 100 g for 15 s.
24. Using a vacuum manifold or centrifugation, wash the Hz beads 4 times with 500 μL of Trypsin Digest Solution (50 mM AMBIC-15% ACN) if digesting with Trypsin or with 500 μL of 50 mM AMBIC if digesting with Chymotrypsin.

Protease digest and washes:

25. After removing the last wash, plug the bottom of the column.
26. Add 1:100 w/w of sequencing grade trypsin (*i.e.* add 10 μg trypsin for processing 1 mg of cell lysate) diluted in 500 μL of Digest Solution or add 1:100 of chymotrypsin diluted in 500 μL of 50 mM AMBIC to the Hz beads.
  *Note: Using both trypsin and chymotrypsin in separate digestion of the same sample is optional, but generally improves the number of identifications. If only a single protease is being used, trypsin is the best choice.*
27. Cap the top of the column and apply parafilm around the plug and also the cap to prevent leakage.
28. Invert a few times to re-suspend the Hz beads and place the spin column in a labeled 1.5 mL tube.
29. Place the sample in the head over head tube rotator and use parafilm again to secure the sample on the tube holder of the rotator.
30. Place the tube rotator in the incubator set at either 37 °C for trypsin digestion or at 25 °C for chymotrypsin digestion for an O/N digestion (12 to 18 h).
31. Place the column into a new 2 mL tube and centrifuge at 100 g for 15 s.
  *Note: The flow through contains the protease-released peptides which can be frozen at −80* °*C and analyzed by Mass Spec separately. However, we find that this fraction is often not cell surface specific.*
32. Transfer the column in a new 2 mL tube and remove the remaining non-glycosylated peptides with a series of washes with salts and organic solvents using a Vacuum manifold or by centrifugation as described previously.
33. Wash the Hz beads 3 times with 500 μL of 1.5 M NaCl and then 3 times with 500 μL of Organic Wash Solution (60% ACN-0.1% TFA) and 3 times with 500 μL of MeOH.
34. Finally, wash the Hz beads 6 times with 500 μL of 50 mM AMBIC to completely remove the organic solvents.

Release of N-linked glycopeptides with PNGase F:

35. Using a widened tip, add 500 μL of 50 mM AMBIC to the Hz beads and transfer them from the spin column into a new labelled 1.5 mL tube, if necessary.
36. Spin the tube for 1 min at 1500 g and then very carefully pipet out the AMBIC buffer without disturbing the bed of Hz beads.
37. Add 100 μL of 50 mM AMBIC containing 4 units of PNGase F to the Hz beads.
  *Note: 4 units of PNGase F is typically used when processing an input of 1* *mg of cell lysate. However, we've noticed some variability in the activity (units/mg) of PNGase F from lot to lot. If the number of N-linked peptide are lower than expected OR if the amount of intact protein (PNGase F) is high in the sample leading to contamination of the LC system, the amount of PNGase F should be adjusted accordingly.*
38. Gently flick the tube to re-suspend the Hz beads and then incubate O/N (12 to 18 h) at 37 °C without rotation.
*Note: Since the sample volume is small, there is no need for rotation.*
39. Transfer the supernatant that now contains the released N-linked glycopeptides to a new 1.5 tube.
40. To maximize peptide recovery, add 100 μL more of 50 mM AMBIC to the Hz beads, flick the tube a few times to re-suspend the beads, let the beads settle then pool the supernatant with the previously collected N-linked glycopeptides.
41. Repeat the previous step one more time.
42. Pre-wet a new mini-spin column with 400 μL of 50 mM AMBIC by centrifuging it for 1 min at 100 g inside a 2 mL tube.
43. Remove any leftover Hz bead particles from the released N-linked glycopeptides fraction by spinning the sample through the pre-wetted column into a new 1.5 mL tube for 1 min at 100 g.
*Note: We analyze the N-linked fraction without any additional clean-up step. If a C18 cleanup step (e.g. stage tip) is incorporated prior to mass spectrometry analysis, this step may not be required.*
44. Speed vac the sample down to concentrate the peptides.
  *Note: for an input of 1* *mg of oxidized cell lysate, the sample is typically brought down to a volume of about 30 to 60* μ*L.*
45. Freeze the sample at −80 °C until ready to analyze.

**Notes on mass spectrometry (MS) and data analysis:** While this protocol is focused on the sample preparation side for cell surface proteomics, we have identified some important considerations when doing MS analysis of these samples, as outlined below:

• In our experience, only the fraction of peptides released by PNGaseF are specific for cell surface proteins. The peptides released by trypsin or chymotrypsin are highly enriched in cell surface proteins, but also contain many non-membrane proteins due to non-specific binding.
• It is important to include deamidation of asparagine as a variable modification during the database search to identify peptides that have had a glycan removed by PNGaseF.
• For maximum specificity for cell surface proteins, it is important to filter peptide identifications to require the presence of a deamidated N-linked glycosylation consensus site (N-X-S/T/C where X is any amino acid except P) when producing the final peptide identification list.
• When using chymotrypsin, a less specific peptide search is required. It is important to take special care with managing false-discovery rates, as these can be inflated with the more open database search.
• In general, analysis of cell surface peptides is improved by MS acquisition methods that are focused on sensitivity of detection, rather than speed of MS2 acquisition.

**Method validation:** To exemplify the method above, we performed cell surface proteomics on three adherent human tumor cell lines (A549, OVCAR3, and U87MG). The resulting peptides were analyzed by nanoLC-MS on a Thermo Scientific QE-HX and peptides were identified using a search against the human Uniprot database using MSFragger [4,7] as implemented in Fragpipe. Peptides and proteins identified in each sample are listed in Supplementary Table 1. Additional methodological details are available in the supplementary methods.

**Fig. 1:**
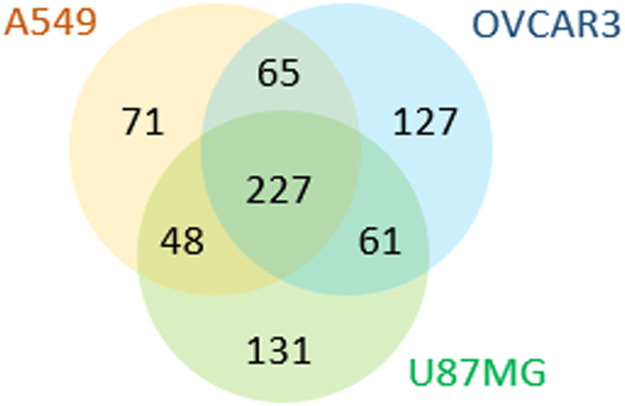
Proteins identified bu D-CSC in three all lines.

The results of the D-CSC method with each of the three cell lines are highlighted in Table 1. In each cell line, between 411 and 467 unique proteins were identified from over 2000 unique N-glycopeptides. In total, 730 proteins were identified in the three cell lines. Only 227 of these were found in all 3 cell lines, while 329 proteins were only found in 1 of the 3 cell lines (Fig. 1). To confirm that cell surface proteins were being identified, we compared the proteins identified in the tumor cell lines to three different published lists of human cell surface proteins: (1) Proteins present in the Human Cell Surface Protein Atlas (CSPA) [2], (2) Proteins predicted to be cell surface by SURFY, a machine learning predictor for human cell surface proteins [1], and (3) Proteins present in a cell surface filter published in Sobotzki et al. as Supplementary Data 3 [5]. As can be seen in Table 2 between 76% - 94% of the proteins identified in each of the cell lines using the protocol above were considered cell surface, depending on the list of defined cell surface proteins as a baseline. Alignment of the data from our 3 cell lines to various Uniprot and GO ontology terms that are associated with cell surface proteins is shown in Supplementary Table 2. For comparison, we have also calculated the percent of proteins in the CSPA (which were all identified by the original CSC method) that are associated with these same Uniprot and GO ontology terms. In nearly all cases, the data generated by D-CSC on the three tumor cell lines compares favorably with the proteins identified by the original CSC method in terms of cell-surface selectivity.

**Table 1:** Characteristics of cell surface glycopeptides and proteins identified using the D-CSC method in three tumor cell lines.

Cell Line	# Unique peptides	# Unique N-glycopeptides (contains N-glyco consensus seq)	# Unique N-glycosites	# Unique Proteins	% Cell Surface Proteins as defined by CSPA [2]	% Cell Surface Proteins as defined by SURFY [1]	% Cell Surface Proteins as defined by Sobotzki et al. [5]
A549	2312	2193 (95%)	1124	411	78%	82%	94%
OVCAR3	2490	2310 (93%)	1228	480	79%	77%	93%
U87MG	2450	2331 (95%)	1161	467	81%	76%	93%

**Table 2:** Comparison of published CSC data with this direct cell surface hydrazide capture (D-CSC) method for U87MG cell surface datasets.

Cell surface proteins from U87MG	# proteins *	# (%) cell surface proteins as defined by CSPA	# (%) cell surface proteins as defined by SURFY	# (%) cell surface proteins as defined by Sobotzki et al. [5]
Proteins identified in both CSC and d-CSC	209	192 (92%)	170 (81%)	197 (94%)
Proteins identified only in published CSC study	164	149 (91%)	62 (38%)	109 (66%)
Proteins identified only in this analysis (D-CSC)	258	186 (72%)	184 (71%)	237 (92%)

To confirm that the specificity of the simplified cell surface capture method described above is similar to the previously published CSC technology [8], we compared the published CSC results from U87MG cells [2] to our data from this same cell line. Since it is not meaningful to compare the numbers of proteins identified in each of these studies due to differences in mass spectrometry instrumentation and data analysis pipelines, we focused our comparison on the percent of proteins that were predicted to be cell surface in each dataset as determined by comparison with the same three lists of cell surface proteins described above. The results of this analysis are shown in Table 2. Of the 631 proteins identified in either study, 209 proteins were identified in both our D-CSC analysis and in the published CSC analysis. As expected, these proteins show a high degree of specificity for predicted cell surface proteins. Of the proteins identified only in one of the two analyses, the D-CSC method described here identified a higher percentage of cell surface proteins as defined by SURFY [1] or *Sobotski* et al. [5]. The original CSC method identified a higher percentage of cell surface proteins as defined by the human Cell Surface Protein Atlas (CSPA). This finding was not unexpected since the CSC analysis of U87MG cells analyzed here was one of the datasets used to build the CSPA. Taken together, these results suggest that we did not lose significant cell surface specificity by eliminating the biotinylation step and that it is possible that cell surface selectivity was improved when using D-CSC.

D-CSC has some minor advantages over the original CSC protocol. It is often practical for the cell surface labeling step to be done by collaborators who are experts in growing the cells of interest. In D-CSC, these collaborators can simply oxidize the cells as outlined in the “Glycan oxidation of cell surface proteins” section, freeze the cell pellet, and ship it to the mass spectrometry lab for sample processing. The glycan oxidation steps in D-CSC require only inexpensive reagents that can be easily purchased by the collaborating lab. It uses methods that are very familiar to any scientist with tissue culture expertise and does not require chemical labeling with a specialized biotin reagent like CSC. The second advantage of D-CSC is that it enables analysis of the protease-released fraction. While this fraction is not specific to the cell-surface, it is enriched in cell surface proteins and can provide interesting protein identifications in comparative proteomic experiments that are not seen in whole cell peptide digests or in the cell surface profiling. This would include surface glycoproteins with only o-linked glycosylation or that have N-linked glycosylation sites that fall in peptides that are not amenable to MS identification. In CSC, the protease-released fraction is generally contaminated with abundant streptavidin peptides which can limit loading and lead to contamination of nano-LC systems, while in D-CSC this fraction can be more easily analyzed with optimal loading to increase identifications.

## CRediT authorship contribution statement

**Tammy-Lynn Tremblay:** Methodology, Investigation, Writing – original draft, Writing – review & editing, Visualization. **François Fauteux:** Methodology, Validation, Formal analysis, Data curation, Writing – review & editing. **Deborah Callaghan:** Investigation, Writing – review & editing. **Jennifer J. Hill:** Conceptualization, Methodology, Formal analysis, Visualization, Writing – original draft, Writing – review & editing, Supervision.

## Declaration of Competing Interests

The authors declare that they have no known competing financial interests or personal relationships that could have appeared to influence the work reported in this paper.

## Data Availability

Data will be made available on request.

## Appendix Supplementary materials

Image, application 1

Image, application 2
