# Supplementary material for: Cell surface profiling of cultured cells by direct hydrazide capture of oxidized glycoproteins
Source: MethodsX. 2023 Aug 26;11:102349. doi: 10.1016/j.mex.2023.102349 (PMC10480300; doi:10.1016/j.mex.2023.102349)
Supplement: Supplementary file 2 [file mmc2.docx]

**Supplementary Table 2: Comparison of the % identified proteins in each category between D-CSC data from 3 cell lines and the Cell Surface Protein Atlas (CSPA) which was built using the CSC method.**

| Source | Term | CSPA (1443 prot) | A549:  D-CSC  (411 prot) | OVCAR3: D-CSC (480 prot) | U87MG: D-CSC (467 prot) | All D-CSC IDs (730 prot) |
| --- | --- | --- | --- | --- | --- | --- |
| Uniprot: Subcellular localization | Cell membrane | 40.6% | 55.0% | 52.9% | 49.0% | 49.3% |
| Uniprot: Subcellular localization | Cell surface | 1.1% | 1.0% | 1.9% | 1.7% | 1.6% |
| Uniprot: Topology | GPI-anchor | 3.9% | 6.3% | 6.0% | 5.1% | 5.9% |
| Uniprot: Topology | Multi-pass membrane protein | 22.9% | 30.7% | 29.6% | 28.3% | 28.9% |
| Uniprot: Topology | Single-pass membrane protein | 48.0% | 55.0% | 54.2% | 53.3% | 51.4% |
| Uniprot: Domains | Transmembrane region | 70.8% | 85.2% | 83.3% | 81.4% | 80.0% |
| Uniprot: Domains | Extracellular | 53.3% | 73.0% | 70.6% | 66.6% | 66.2% |
| GOA | plasma membrane (GO:0005886) | 55.0% | 73.0% | 67.9% | 66.6% | 65.5% |
| GOA | external side of plasma membrane (GO:0009897) | 7.4% | 8.8% | 8.1% | 8.8% | 7.0% |
| GOA | cell surface (GO:0009986) | 15.5% | 24.6% | 23.3% | 21.8% | 20.3% |

**Supplementary Methods**

*Cell culture methods*

A549 and OVCAR3 adherent cells were grown in Corning® 150 mm TC-treated Culture Dish using RPMI culture media supplemented with 2 mM L-glutamine and 10% FBS. U87MG adherent cells were grown in Corning® 150 mm TC-treated Culture Dish using DMEM culture media supplemented with 2 mM L-Glutamine and 10% FBS. All cells were grown until they reach between 80 and 100% confluency.

*Mass spectrometry and data analysis*

N-linked peptide fractions were analyzed by LC-MS/MS on a Q-Exactive HF mass spectrometer using a 150 µm C18 LC column and a 70 minute gradient. MS spectra were collected at 60k resolution with data-dependent MS2 on the top 15 ions at 15k resolution and a 30s dynamic exclusion time. Raw MS data files were searched with MSFragger 3.0 as implemented in FragPipe v13.0 using the built-in configuration for “Nonspecific-peptidome” which identifies peptides with a 1% FDR with the following adjustments: Database = Uniprot human database (from 10 June 2020) with decoy sequences added by Philosopher 3.2.7; Variable modifications: oxidation (M, max = 1), deamidation (N, max = 3); peptide length: 7 - 40; peptide mass range: 800-8000. A split database (15 slices) was used to overcome memory limitations.

Using a Python script, the MSFragger output file “peptide.tsv” was further filtered to keep only peptides that contained at least one deamidated Asn and at least one Asn in an N-linked consensus sequence (NxT, NxS, or NxC, where x is any amino acid except P).  A protein was considered identified if at least one unique N-linked peptide was identified, leading to a protein FDR of <1%. Protein FDR was calculated based on remaining decoys after all filtering steps.

*Comparison with published lists*

For all lists, Uniprot accession numbers were converted to current HGNC gene symbols (Uniprot June 2021 download) and duplicate HGNC gene symbols were deleted from each list. The CSC data for U87MG cells was extracted from S1 file – Table B published in Bausch-Fluck *et al*. *PLoS One* **2015**, *10*, 1–22, doi:10.1371/journal.pone.0121314.
